# Acknowledgment to reviewers—November 2019 to October 2020

**DOI:** 10.1080/20961790.2020.1848411

**Published:** 2020-12-28

The Editors of the *Forensic Sciences Research* (*FSR*) wish to thank the following people, and any other reviewer whose name has been inadvertently omitted, for giving your time and expertise to review papers and facilitate the smooth running of *FSR* between November 2019 and October 2020.

Julia S. Allwood, *USA*

Corry Azzopardi, *Canada*

Eric J. Bartelink, *USA*

Jean-Pol Beauthier, *Belgium*

William R. Belcher, *USA*

Sompop Bencharit, *USA*

Einar Bjorgo, *Switzerland*

Katherine Brown, *UK*

Joshua L. Brunty, *USA*

Francesco P. Busardò, *Italy*

Kirsten Buße, *Switzerland*

Xiaohong Chen, *China*

Yao Chen, *Italy*

Caroline Chimeno, *Germany*

Danilo De Angelis, *Italy*

Mario A. de Oliveira, *Brazil*

Fabrice Dedouit, *France*

Tania Delabarbe, *France*

Yann Delannoy, *France*

Matt DeLisi, *USA*

Zhenhua Deng, *China*

Jessirie Dilag, *Australia*

Denise Donlon, *Australia*

Gary Edmond, *Australia*

Sarah Ellingham, *Switzerland*

Audrey Farrugia-Jacamon, *France*

Gabriel M. Fonseca, *Chile*

Alex Forrest, *Australia*

Christine A. M. France, *USA*

Ademir Franco, *Portugal*

Shanlin Fu, *Australia*

Laura C. Fulginiti, *USA*

Ivan Galić, *Italy*

Matteo D. Gallidabino, *UK*

Binita Gandhi, *India*

Imanol Garamendi, *Spain*

Rodrigo Garrido, *Brazil*

Dominic Gascho, *Switzerland*

Sara M. Getz, *USA*

Cláudia Gomes, *Spain*

David G. Greenhalgh, *USA*

Sameera A. Gunawardena, *Sri Lanka*

Fei Guo, *China*

Hong Guo, *China*

Yucheng Guo, *China*

Yadong Guo, *China*

Bhupendra Gupta, *India*

Mukaddes Gürler, *Turkey*

Julia Halo, *USA*

Edward Harshany, *USA*

Lyndal Henden, *Australia*

Christopher H. Hendon, *USA*

Chong Chin Heo, *Malaysia*

Yazmín Hernández-Díaz, *Mexico*

Michael Hlastala, *USA*

Yiping Hou, *China*

Shannon C. Houck, *USA*

Rachel Houston, *USA*

Honghua Hu, *Australia*

Daixin Huang, *China*

Ping Huang, *China*

Kazuhiko Imaizumi, *Japan*

Victor Jacometti, *Brazil*

Adam Jewiss-Gaines, *Canada*

Kaveh Jorabchi, *USA*

Neeti Kapoor, *India*

Beytullah Karadayi, *Turkey*

Jessica M. Karanian, *USA*

Shakeel Kazmi, *Pakistan*

Lay S. Khoo, *Malawi*

Ardavan Khoshnood, *Switzerland*

Hilton Kobus, *Australia*

Dmitry M. Kolpashchikov, *USA*

Robert Kronstrand, *Sweden*

Barry K. Lavine, *USA*

Loong Chuen Lee, *Malaysia*

Biao Li, *China*

Lili Li, *China*

Zhen Li, *China*

Zhengdong Li, *China*

Shiquan Liu, *China*

Xiling Liu, *China*

Federico Lugli, *Italy*

Haibo Luo, *China*

Teresa Magalhães, *Portugal*

Salvatore A. E. Marras, *USA*

Luis J. Martinez-Gonzalez, *Spain*

Kristy A. Martire, *Australia*

Britny Martlin, *Canada*

Jennifer M. McKinley, *Ireland*

Kelly A. Meiklejohn, *USA*

Liang Meng, *China*

Jean-François Michard, *France*

Arjan Mieremet, *Netherlands*

Carole Miossec, *France*

Tawachai Monum, *Thailand*

Geoffrey S. Morrison, *UK*

Brent L. Nielsen, *USA*

Alexandrina S. Nikova, *Greece*

Gérard Niveau, *Switzerland*

Emilio Nuzzolese, *Italy*

Jake W. O'Brien, *Australia*

Okorie Okorocha, *USA*

Ricardo Orlando, *Brazil*

Morgan Philp, *Australia*

Noemí Rivaldería, *Brazil*

Kim Roberts, *Canada*

Markus A. Rothschild, *Germany*

Maria Saiz, *Spain*

Emmanouil I. Sakelliadis, *Greece*

Roberto Scendoni, *Italy*

Yu Shao, *China*

Inga Siebke, *Switzerland*

Carolina S. Silva, *Brazil*

Heitor Simoes Dutra Correa, *Italy*

Nancy Singla, *India*

B. Holly Smith, *USA*

Kuan Sun, *China*

Qiran Sun, *China*

Petra Švábová, *Slovakia*

Krzysztof Szpila, *Poland*

María J. Tabernero, *Spain*

Ruiyang Tao, *China*

Ian J. Turner, *UK*

Joost van Beusekom, *Germany*

Rick R. van Rijn, *Netherlands*

Jozue Vieira Filho, *Brazil*

Maria Isabel de Oliveira e Britto Villalobos, *Brazil*

Qi Wang, *China*

David Webb, *USA*

Sandy K. Wurtele, *USA*

Jianhui Xie, *China*

Hui Yan, *China*

Jun Yao, *China*

Tarek Younis, *UK*

Tao Yu, *China*

Clement C. Zai, *Canada*

Francesco Zampa, *Italy*

Jinhua Zeng, *China*

Lagabaiyila Zha, *China*

Jian Zhang, *China*

Qinting Zhang, *China*

Suhua Zhang, *China*

Xinqing Zhang, *China*

Axel Zinkernagel, *Germany*

